# 33% hydrogen peroxide as a Neoadjuvant treatment in the surgical excision of non-melanoma skin cancers: a case series

**DOI:** 10.1186/s40463-020-00433-6

**Published:** 2020-06-01

**Authors:** N. Mundi, K. Jordan, P. Doyle, C. Moore

**Affiliations:** 1 Department of Otolaryngology – Head and Neck Surgery, London Health Sciences Centre, Victoria Hospital, University of Western Ontario, 800 Commissioners Road E, London, ON N6A 5W9 Canada; 2 Departments of Oncology and Biophysics, University of Western Ontario, London, Ontario Canada; 3 Division of Facial Plastic and Reconstructive Surgery, Department of Otolaryngology – Head and Neck Surgery, University of Western Ontario, London, Ontario Canada; 4 Division of Surgical Oncology, Department of Oncology, University of Western Ontario, London, Ontario Canada

**Keywords:** Skin neoplasms, Peroxide

## Abstract

**Background:**

Hydrogen peroxide (H_2_O_2_) is a product of respiration in mitochondria and an important oxidizing agent in biological systems. Previous investigations have shown the efficacy of H_2_O_2_ in treating skin conditions such as seborrheic keratosis and actinic keratosis. In an area like the face, reconstruction of excision defects and ultimately aesthetic outcomes are of utmost importance. Hydrogen peroxide may represent a simple yet effective method at shrinking non-melanoma skin cancers (NMSC) of the head and neck before they are excised.

**Methods:**

Eleven consecutive patients presenting to our cutaneous malignancy clinic had their skin lesions evaluated by the senior author for participation in the study. Lesion length and width was measured. Hydrogen peroxide formulated at a concentration of 33% was rubbed into the lesion until blanching was observed. Lesions were re-measured at follow up. Excisional biopsy was then performed and histopathological diagnosis was obtained. Statistical analyses compared pre- and post-treatment lesion dimensions.

**Results:**

Seventeen biopsy-proven NMSC lesions were included in this investigation. Statistically significant reductions in the length (*p* < 0.001) and width (*p* < 0.001) were observed with H_2_O_2_ treatment. For some lesions, H_2_O_2_ was the sole treatment required, with post-treatment biopsy demonstrating no evidence of malignancy. Patients endured minimal discomfort during treatment and no long-term side effects were observed. Follow up at 6 months revealed no recurrences.

**Conclusions:**

We have demonstrated a significant reduction in the size of multiple lesions after application of 33% hydrogen peroxide, simplifying definitive excision and reconstruction. Hydrogen peroxide demonstrated an ability to successfully treat non-melanoma skin cancers as well.

## Background

Non-melanoma skin cancers (NMSC) are the most commonly occurring cancers worldwide. The incidence of these malignancies is steadily rising secondary to the advancing age of the general population as well as sun exposure. Gold standard treatment modalities for NMSC include surgical excision, photodynamic therapy and radiation therapy. Each of these is accompanied by risks to the patient including pain, bleeding, infection, scarring and pigment alterations. Patients with NMSC of the head and neck may be particularly concerned regarding these risks as this region is cosmetically sensitive.

Hydrogen peroxide (H_2_O_2_) is a product of respiration in mitochondria and an important oxidizing agent in biological systems. In dermatology, it is used frequently as a topical antiseptic and hemostatic agent [1]. These effects are generally achieved with application of dilute hydrogen peroxide (typically 3%) to the skin, with little to no side effects for patients besides mild discomfort. As it is a potent oxidizing agent, hydrogen peroxide can exert a role in oxidative stress, although the exact mechanism through which this occurs is not yet known. Giulivi and Davies propose that H_2_O_2_ may interact with hemoglobin in the dermal capillaries, producing oxidized forms of hemoglobin such as ferrylhemoglobin which is highly reactive [2]. It is, therefore, possible that H_2_O_2_ could cause necrogenous oxidation and oxygen-induced apoptosis of cells in non-melanoma skin cancers.

A previous investigation examining the use of H_2_O_2_ to treat seborrheic keratosis at or above a concentration of 23% found that the mean number of benign epidermal proliferations remaining at 90 days after treatment was significantly lower in the H_2_O_2_ group compared to placebo; this was after an average of six applications of hydrogen peroxide at roughly 1 week intervals. In that particular investigation, the only side effect from application of the H_2_O_2_ solution was less than 10 min of “burning” at the application site [3]. Recently, the Food and Drug Administration has approved hydrogen peroxide 40% topical solution for the treatment of seborrheic keratoses [4]. In melanoma, hydrogen peroxide may also have a beneficial effect. Fang et al. found that H2O2 was an effective radiosensitizer in a radioresistant melanoma cell line. Specifically, H2O2 synergized with radiotherapy causing increased expression of p15, and reduced expression of cyclin D, cyclin-dependent kinase 2 and 4 [5].

Hydrogen peroxide also has been used in combination with other topical treatments such as NSAIDs to successfully combat precancerous lesions such as actinic keratosis [6]. Additionally, H_2_O_2_ may be advantageous as part of photodynamic therapy protocols to treat non-melanoma skin cancers [7, 8]. Currently, surgical excision remains the gold standard treatment for non-melanoma skin cancers, with recommended margins being 0.5–1.0 cm depending on the specific pathology of the lesion itself.

In an area like the face, reconstruction of excision defects and ultimately aesthetic outcomes are of utmost importance. Particularly large skin lesions which must be excised can sometimes necessitate rotational flaps or even skin grafting, each of which has disadvantages in terms of healing and scarring. Given its relatively benign nature and previous efficacy in treating other skin growths, hydrogen peroxide may represent a simple yet effective method at shrinking non-melanoma skin cancers of the head and neck before they are excised. In doing so, it could minimize the invasiveness of surgical excision, thereby accelerating healing and giving patients better aesthetic outcomes. Moreover, this technique addresses the weaknesses of hydrogen peroxide alone by providing histologic diagnosis and pathologic margins of the lesion with complete primary excision. The objective of this investigation is to determine the effectiveness of hydrogen peroxide 33% topical solution (HP33) as a neo-adjuvant treatment for non-melanoma skin cancers undergoing surgical excision.

## Methods

### Study design

This investigation was a prospective, single-institution case series (HSREB 110641). The target patient population included individuals with histologically confirmed non-melanoma cervicofacial cutaneous malignancy referred to our tertiary care skin cancer clinic for definitive management. In order to avoid any direct selection bias, we elected to examine consecutive patients referred to our center for eligibility and who met all inclusion and exclusion criteria. From the sample series who participated, we accrued patients over a three-month period. After intervention, patients were followed for 6 months.

### Inclusion and exclusion criteria

Histologically confirmed cutaneous malignancies included in this study were squamous cell or basal cell carcinoma, recurrent squamous or basal cell carcinoma occurring in previously radiated areas, and in-situ squamous cell or basal cell carcinoma. Patient characteristics included the ability to tolerate intended treatment, no prior treatment with investigational agents, age greater than or equal to 18 years, and life expectancy estimated at greater than 4 years.

Exclusion criteria for this investigation include patients with a history of cutaneous photosensitization, porphyria, hypersensitivity to porphyrins or photodermatosis. In addition, use of the following drugs prior to entry into the study was grounds for exclusion: topical medications, corticosteroids, antimicrobials, alpha-hydroxy acids (lactic acid), retinoids (Retin-A). Also, systemic therapy with steroids, topical application of 5-FU, masoprocol (Actinex), systemic treatment with retinoids (Tegison, Accutane), chemotherapeutic agents, immunotherapy or photosensitizing drugs.

Those individuals deemed appropriate for entry into the study were given a detailed information package outlining the study procedures, as well as the potential risks and benefits of treatment. Following this, informed consent for participation was obtained.

### Documentation of lesions and application of HP33

All individuals included in the investigation had their lesions evaluated by the senior author for participation in the study. The senior author (CM) is a Facial Plastic surgeon with 15 years of experience practicing in a high-volume tertiary care center. The borders of the lesion were outlined directly on the patient’s skin using a standard surgical felt-tip marking pen under 3.5x loupes (Staedtler Lumicolour permanent marker, size medium, Art. No. 317–3). The lesion borders were then transcribed onto a clear acetate film. Measurements of the length and the width of the lesion were taken in millimeters. Lesions were then photographed before and during intervention.

**Fig. 1:**
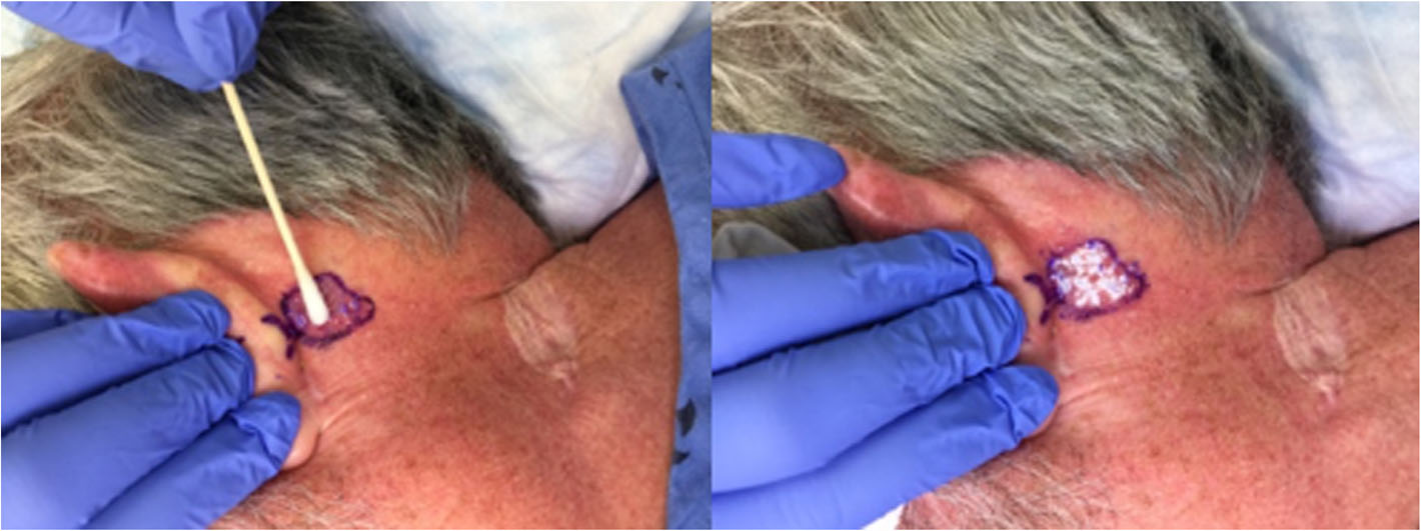
Blanching of skin observed after application of 33% hydrogen peroxide solution

A sterile 70% Isopropyl alcohol prep swab was used to remove keratinous debris and oils from the skin overlying the lesion. A standard cotton tip applicator was soaked in HP33 until wet and used by the senior author to rub HP33 into the lesion until blanching of the lesion was observed (Fig. 1). During application, subjects were asked to describe any sensations experienced to the treatment area. Patients were not given any specific post-intervention instructions.

### Patient follow-up

Patients were then seen 4 weeks post-treatment in a follow-up clinic for assessment of re-demarcating of the lesion with repeat measurements of width and length. If the lesion was amenable to primary surgical excision, the area of excision was anesthetized, and the lesion was resected with primary closure by the senior author. If no visible lesion was present, a 4 mm punch biopsy of the area in which the lesion was previously visible was performed. Patients were seen in follow-up once more 6 months post-treatment for examination of their treatment area.

### Primary outcome

The primary outcome of interest in this investigation was the change in length and width of the target lesion pre- and post-treatment with HP33.

### Data collection and analysis

Data were recorded for various patient, tumour, or treatment factors that may potentially influence the outcome of combined HP33 and surgical therapy. These included patient age and sex, lesion location, and histopathology. The length and width of the lesions was measured pre- and post-treatment with hydrogen peroxide. Pre- and post-treatment lesion dimensions were compared using two-tailed Student’s T tests, with a *p* value less than 0.05 considered to be statistically significant. Knowing that our inclusionary and exclusionary criteria may have limited the number patients who might serve as participants, we did carefully consider sample size. Based on sample size calculation, we determined that a total sample size of 11 individuals, or in this case lesions, was sufficient to detect the hypothesized effect (r ^2^ = .25) 83.0% of the time using a .05 alpha level.

## Results

### Patient population

Twenty lesions were initially included in this investigation, derived from 12 consecutive subjects, who all met inclusion criteria. Three lesions were excluded from analysis as they underwent punch biopsy instead of definitive excision post-treatment with HP33. Therefore, 17 lesions were included for statistical analysis.

The mean age of subjects was 77 years. Biopsy-proven squamous cell carcinoma comprised 18% of lesions and the remaining 82% were basal cell carcinoma. The details regarding lesion location and size is shown in Table 1.

**Table 1:** Patient Characteristics

Subject Number	Sex	Age	Initial Pathology	Site	Length (mm)	Width (mm)
1	Male	67	BCC	Upper Back	20	15
2	Female	71	BCC	Anterior Scalp	16	15
3	Female	82	BCC	R Alar Rim	4	5
				R neck	10	6
				Upper Back	8	8
4	Male	87	BCC	R shoulder	10	3
				R neck	10	10
				L arm	8	10
5	Male	86	SCC	L Temple	28	39
			BCC	R Cheek	7	8
6	Female	64	SCC	L Forehead	25	27
				L Cheek	28	21
7	Male	73	BCC	Right Antihelix	13	10
8	Male	69	BCC	R Nasal Sidewall	8	5
9	Female	84	SCC	L Temple	38	30
10	Female	92	BCC	Nose	21	28
11	Female	74	BCC	Forehead	13	15

### Hydrogen peroxide significantly reduces the size of NMSC

**Fig. 2:**
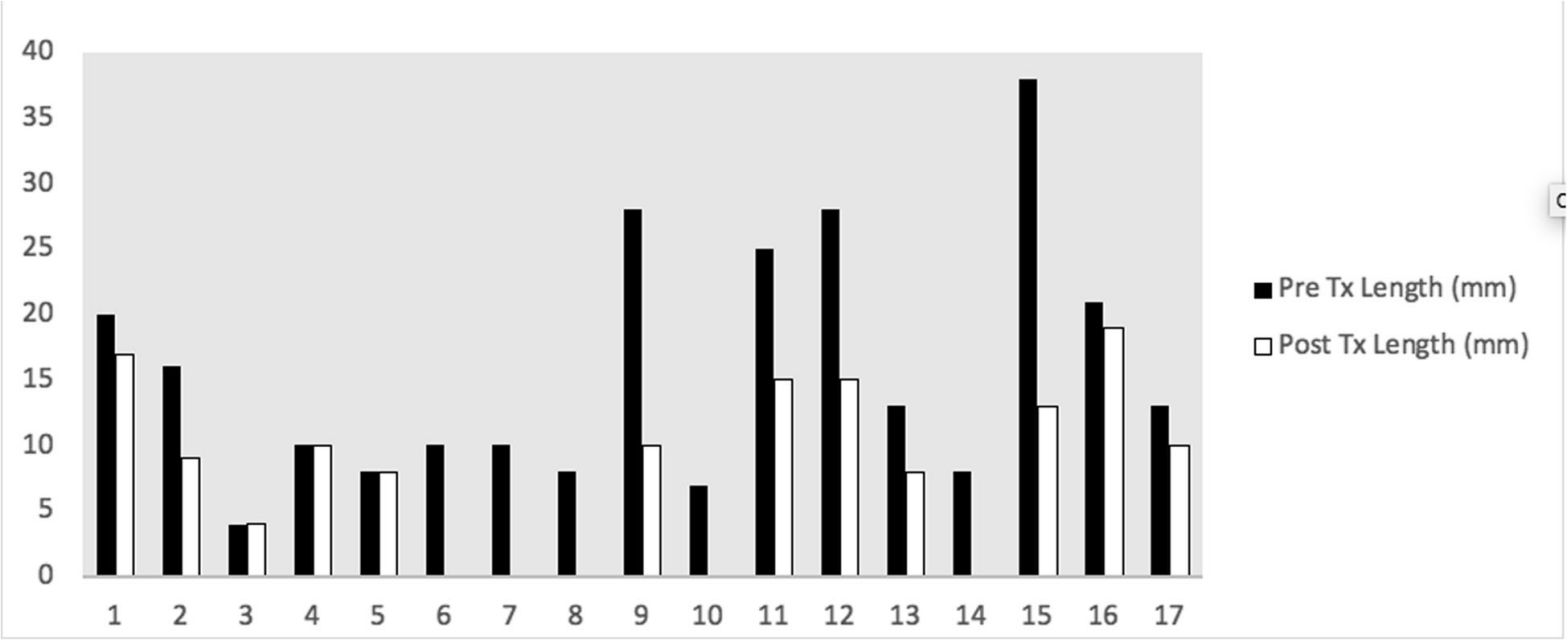
Lesion length pre- and post-treatment with HP33

**Fig. 3:**
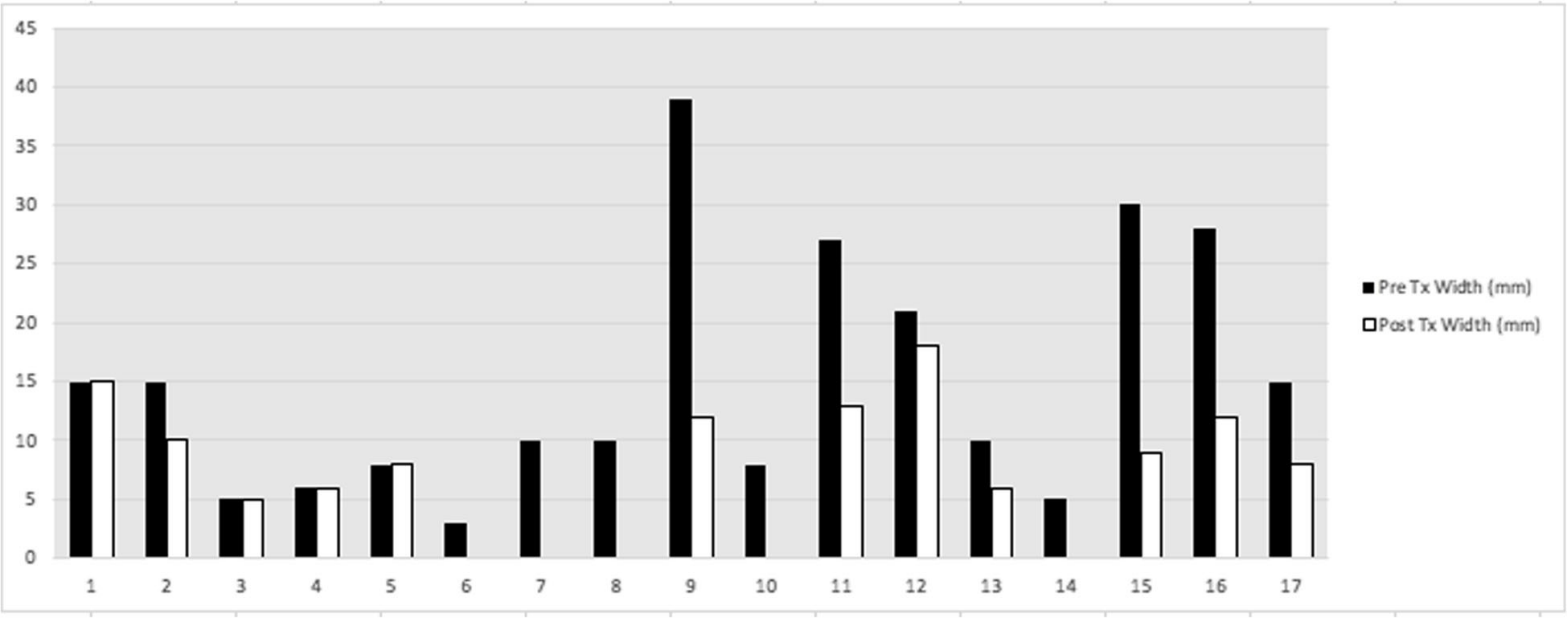
Lesion width pre- and post-treatment with HP33

All lesions included in this study were reduced in terms of their dimensions after treatment with HP33. Specifically, the length of lesions was reduced by a mean of 50%, while the width was reduced by a mean of 48%. Statistical analysis revealed a statistically significant reduction in both length (*p* < 0.001) and width (*p* < 0.001) (Figs. 2, 3). In fact, five basal cell carcinomas were no longer visible under high-powered loupes after treatment with HP33. It is important to note the consistency in the reduction of lesion size for both length and width, a finding that supports the applicability of our treatment. In the case of nine lesions, definitive excision of the previously biopsy-proven NMSC returned as being negative for malignancy with clear surgical margins; this occurred in both basal cell and squamous cell carcinomas. Excision of several lesions was simplified following application of HP33 and were performed using primary closure whereas initial excision would have necessitated rotation flap closure.

Patient discomfort during treatment was self-described by some patients as a slight tingling sensation to the treatment area. Discussion with patients in the post-treatment follow up appointment revealed that the blanching observed during treatment lasted approximately 30 min. Following this, the patient’s skin returned to its original state. No subjects were lost to follow-up. Additionally, no subjects included in this study suffered scarring, recurrence, or other post-treatment sequelae at their 6 month follow-up.

## Discussion

Our investigation is amongst the first in the literature to examine the use of topical hydrogen peroxide as a neoadjuvant treatment for non-melanoma cervicofacial cutaneous malignancies. We have conducted a prospective case-series to study whether this novel treatment modality may reduce the size of lesions requiring excision in the cosmetically sensitive area of the head and neck. Accordingly, the product of this work may be viewed as a proof-of-concept investigation that can lead to larger scale evaluation of hydrogen peroxide as a treatment option. With respect to cutaneous malignancies, hydrogen peroxide has been previously described for photodynamic therapy in the treatment of basal cell carcinoma [7, 8]. The mechanism of action in this respect is thought to be through the ability of hydrogen peroxide to increase oxygen concentrations at the treatment site [8].

We observed a statistically significant reduction in the size of multiple skin malignancies including squamous and basal cell carcinoma treated with a single application of HP33. This resulted in simpler excisions and reduced the need for local flap reconstruction and skin grafting. This more conservative approach to treatment was deemed to be an added advantage relative to potentially negative cosmetic changes secondary to treatment. Further, histopathological analysis of 53% of included biopsy-proven basal and squamous cell carcinomas demonstrated complete resolution after treatment with hydrogen peroxide. Follow up of patients for 6 months post-treatment revealed no local recurrences and no scarring. Treatments were well-tolerated with patients experiencing only minimal discomfort during application that quickly subsided; there were no complications from the application of HP33. It is also important to note that this more conservative means of treatment also may serve to reduce patient fears specific to pain or additional side-effects, as well cosmetic consequences of treatment [9]. However, future work that seeks to identify the occurrence and/or severity of potential side effects would be of value. This would include complimentary assessments of quality of life considerations as has been done in prior studies that have assessed photodynamic therapy [10].

Our findings could have broad implications for the treatment of an extremely large and growing population of individuals affected by cutaneous malignancies. Hydrogen peroxide is a relatively inexpensive and readily available substance that is simple to use and store. Its safety profile has been demonstrated in a multitude of investigations [4]. Although, case reports have described skin damage when it is applied in high concentrations to the skin for prolonged periods of time [11]. Further, one-time application of the agent to skin cancers represents an adjuvant treatment modality that would be feasible for a wide variety of health care professionals including surgeons, dermatologists and primary care physicians to administer.

Several limitations of this study are recognized and worth discussion. Firstly, the sample size we have accrued is relatively small, containing 17 lesions. Concerns related to sample size in the context of otolaryngology have been outlined in the literature and the associated practical limitations of what comprises a “meaningful” change have been discussed [12]. But in seeking to initiate this project, we sought to balance potential patient recruitment limitations along with the desire to increase our confidence in the data gathered. Thus, while small, our sample (*n* = 17 lesions) did exceed calculated sample expectations.

Our study lacked a control group and as such the applicability and external validity of our findings are restricted. However, our intention was to perform a study as a proof-of-concept for hydrogen peroxide. Indeed, the results we have presented are quite dramatic and demonstrate the potential utility of this novel neoadjuvant treatment. However, we have not yet interrogated the mechanism of action of hydrogen peroxide in the treatment of cutaneous malignancy.

We also acknowledge that as larger patient samples are studied, the ability to identify key factors and potential predictors of treatment outcome will emerge; the findings of such follow-up investigations may delimit critical factors that may then optimize the selection of patients, in addition to defining the impact of treatment on lesion area. Finally, the effect of multiple applications of HP33 was not examined in the present study; whether a cumulative treatment effect exists is currently unknown. These areas are clearly indicated as valuable areas for future investigation in larger patient samples. But, our assessment of the current data do support the use of hydrogen peroxide as a potentially viable treatment option.

This investigation has raised a multitude of questions that will require further study. As stated above, the exact way in which hydrogen peroxide appears to shrink or completely treat non-melanoma cutaneous malignancy is still unknown. A possible explanation considers the Warburg effect, wherein transformed cells such as those in cutaneous malignancies undergo a shift from oxidative to glycolytic metabolism, rendering cells more susceptible to oxidative stress [13–16]. This could explain hydrogen peroxide’s ability to eliminate transformed cells while leaving normal skin unharmed. In melanoma, hydrogen peroxide has been shown to induce apoptotic or necrotic cell death depending on the concentration of oxidant applied [17].

In addition, while the present data suggest substantial promise, an expanded sample size in the context of a randomized controlled trial comparing hydrogen peroxide to other known topical treatments such as 5-fluorouracil will assist in elucidating the efficacy of this novel treatment. Multiple applications of HP33 could also be studied to determine whether a cumulative effect exists and further to understand the ideal number of applications to yield a clinically significant result. Finally, long-term follow up to monitor for recurrence of lesions treated with hydrogen peroxide will allow us to determine if the complete resolution of lesions that we have observed is sustained. Collectively, the above concerns will offer a fruitful area for continued research into the application of hydrogen peroxide in non-melanoma skin cancers.

## Conclusions

Hydrogen peroxide 33% topical solution shows some potential as a neoadjuvant treatment modality in the surgical excision of non-melanoma skin cancer. In half of cases, a single application was the sole treatment required for both basal and squamous cell carcinoma. The treatment was well-tolerated by patients and caused no long term sequelae. Hydrogen peroxide is a relatively cheap, readily available agent that may represent an exciting, novel treatment modality for the world’s most common cancer that will undoubtedly require further study in order to reveal its true potential.

## Data Availability

The datasets used and/or analysed during the current study are available from the corresponding author on reasonable request.

## Abbreviations

NMSC: Non-Melanoma Skin Cancer
HP33: 33% Hydrogen Peroxide
BCC: Basal Cell Carcinoma
SCC: Squamous Cell Carcinoma
H_2_O_2_: Hydrogen Peroxide
